# The internet is parents' main source of information about psychiatric manifestations of 22q11.2 deletion syndrome (22q11.2DS)^☆^

**DOI:** 10.1016/j.ejmg.2013.05.001

**Published:** 2013-08

**Authors:** Marianne B.M. van den Bree, Gregory Miller, Elizabeth Mansell, Anita Thapar, Frances Flinter, Michael J. Owen

**Affiliations:** a Institute of Psychological Medicine and Clinical Neurosciences, Cardiff University, Cardiff, UK; b School of Psychology, Cardiff University, Cardiff, UK; c Clinical Genetics, Guy's & St. Thomas' NHS Foundation Trust, London, UK

**Keywords:** 22q11.2 deletion syndrome, 22q11.2DS, Velo-Cardio-Facial syndrome, Parental awareness, Physical manifestations, Psychiatric manifestations

## Abstract

With advances in laboratory technology, an increasing number of potentially pathogenic CNVs is recognised. The phenotypic effects of some CNVs are well characterised, however, it remains unclear how much information reaches the parents of affected children and by what route. The 22q11.2 deletion syndrome (del22q11.2) is caused by the deletion of approximately 40 genes from the long arm of chromosome 22 and was first described in 1955 [1]. Our study reports the extent to which parents of an affected child are aware of the various manifestation of the condition and describes how they first learned about these potential problems.

Dear Editor,

With advances in laboratory technology, an increasing number of potentially pathogenic CNVs is recognised. The phenotypic effects of some CNVs are becoming increasingly well-characterised, however, it remains unclear how much information reaches the parents of affected children and by which route.

The 22q11.2 deletion syndrome (22q11.2DS, also known as Velo-Cardio-Facial Syndrome, Shprintzen syndrome and Di George syndrome) is caused by the deletion of approximately 40 genes from the long arm of chromosome 22 and was first described in 1955 [1] (see Ref. [2]).

22q11.2DS is associated with physical problems, developmental delay and psychiatric conditions. The incidence of palatal anomalies is 69–98% [3,4], while heart and kidney defects are reported to occur at 74–82% and ∼37%, respectively [3,4]. Developmental delay is common, including learning disability 99% [4] and speech delay 90% [5]. Psychiatric disorders (reported range 73–80% [6,7]) include obsessive-compulsive disorder (14–33%) [8,9]; psychosis (30%) [10], and schizophrenia (21–24%)[8,10], and depressive (6–18%) [6,7,10,11] and anxiety disorder (44–53%) [6,11].

We aimed to establish the extent to which parents of a child with 22q11.2DS are aware of the various manifestation of the condition and describe how they first learned about these potential problems.

We conducted a survey, via the Bristol Online System (BOS), which was advertised on the website of two UK-based 22q11.2DS support organisations. A paper form of the survey was also completed by parents at the annual conference of one of these organisations in 2010. We opted for recruitment through support organisations rather than clinical settings, because the latter strategy would have presented a more narrow range of parental experiences. Fifty seven parents from the UK & Ireland took part; 7% of whom reported having 22q11.2DS themselves. Their children's reported ages ranged from <1 to 9 years.

Table 1 presents the findings.

Almost all parents (99%) were aware of developmental delay (100% for learning difficulties and 98% for speech delay). Approximately half of the parents who indicated awareness had learned about these issues from clinicians (50% and 55%), either at diagnosis or later. The internet was the most frequently reported other source of information about developmental delay (23–33%).

Most parents (90%) were also aware of physical manifestations associated with 22q11.2DS (palate, heart or kidney defects, range 83–94%); the majority had learned about these from clinicians (62%, range 59–64%), with the internet representing the most frequently reported other source of information (except for heart defects).

With regard to psychiatric manifestations, parental awareness ranged from 69% (obsessions/compulsions) to 85% (psychosis/schizophrenia), with an average over the four conditions of 78%. Here, the most frequently reported source of information was the internet (41–44%). Clinicians were reported as first source of information by between 24% (obsessions/compulsions and anxiety) and 30% (psychosis/schizophrenia) of parents (average 27%).

We examined whether there was a difference in the main source of information (clinicians versus other sources in Table 1) between physical, developmental manifestations, and psychiatric manifestations (McNemar's test for matched pair samples, using exact binomial probability calculations, 2-tailed *p*; http://www.vassarstats.net/propcorr.html).

With regard to physical manifestations, clinicians were more likely to inform parents about palate abnormalities compared with obsessions and compulsions (*p* = .01); psychosis/schizophrenia (*p* = .01); depression (*p* = .01); and anxiety (*p* = .00); as well as about heart or kidney problems, respectively, rather than obsessions/compulsions (both *p* = .00); psychosis/schizophrenia (both *p* = .00); depression (both *p* = .00) and anxiety (both *p* = .00).

Parents were also more likely to have learned about developmental delay through clinicians, compared with psychiatric problems. This was the case for speech delay and obsessions/compulsions (*p* = .01); psychosis/schizophrenia (*p* = .03); depression and anxiety (*p* = .02, *p* = .01) as well as learning difficulties and obsessions/compulsions and anxiety (both *p* = .01); however, the rates for learning difficulties and psychosis/schizophrenia (*p* = .08) and depression (*p* = .08) did not differ, indicating that if clinicians informed parents about learning difficulties, they were also likely to inform them about psychiatric conditions.

Finally, when asked about the main source of information about manifestations associated with 22q11.2DS, 44% of parents reported the internet, 28% clinicians, 20% friends and support groups and 7% books/leaflets.

Our finding of reduced likelihood of divulgence at diagnosis of risk of psychiatric disorders, compared with other manifestations, is in agreement with a study of 41 parents of offspring with 22q11.2DS in the US [12], indicating the generalizability of these findings across different countries and healthcare systems. In the Hercher and Bruenner study, 97% of parents were aware of psychiatric disorders, compared with 78% in the current study, a difference likely to be attributable to the fact that the former study was based on offspring ranging in age from <1 to 38 years, of whom 24% were affected with schizophrenia. The two studies also differ in the reported rates of divulgence of information at diagnosis, which are higher for the US study: risk of psychiatric illness (39% [12], compared with 9–11% in the current study); heart defects (77% versus 50%) and learning difficulties (74% versus 30%), potentially reflecting differences in practice between the US and the UK/Ireland. A considerable number of parents in our survey did, however, indicate receiving this information from clinicians (geneticists or other doctors) at a later time.

Our findings are also in line with a survey by Martin et al. [13] of 54 genetic counsellors, who indicated that during the initial session, they were less likely to discuss psychiatric problems associated with 22q11.2DS, compared with physical health conditions having a later age of onset. The majority of counsellors in this study did nevertheless feel it was important to disclose risk of psychiatric disorder.

We expect that an increasing number of children will be diagnosed with medical conditions caused by CNVs. With this letter we hope to prompt a discussion about the appropriate time and ways to divulge information about specific aspects of genetic syndromes to parents of affected children.

It is difficult to decide when to inform parents of the range of possible manifestations associated with a pathogenic CNV. Around birth, or at the time of diagnosis, physical problems such as a heart defect or cleft lip/palate may represent more pressing matters to discuss than risk of a psychiatric disorder, such as schizophrenia, which is more likely to become a concern once the child reaches adolescence. Providing too much information may be overwhelming and unduly burden parents at an early stage. On the other hand, if this information is not made available, parents may find themselves left to their own devices trying to interpret, understand and cope with changes in their child's behaviour.

The study by Martin et al. [13] conducted interviews with the parents of four adult offspring with 22q11.2DS and schizophrenia. Three indicated they would have preferred to have been informed about psychiatric risks at the time of diagnosis of 22q11.2DS, even if other urgent physical health problems were present. Even though based on retrospective accounts provided by a very small sample of parents whose offspring developed a serious mental health disorder, this study does suggest that psychiatric risks should first be addressed during the early stages of genetic diagnosis. The high rates of childhood psychiatric problems reported for 22q11.2DS as well as the evidence that early diagnosis and treatment can have both short- as well as long-term beneficial impacts [14, 15] provide further support for this approach.

A stepwise approach could be used, where childhood-onset psychiatric problems are first reviewed with the parents during the initial genetic counselling session(s), while later-onset psychopathology is discussed during follow-up sessions. Parents need to be aware that they should contact their family physician (General Practitioner) if they are concerned about their child's behaviour, with the possibility of referral to specialist psychiatric services. By the time the patient reaches later adolescence they should be offered their own counselling session, as part of which the routes they can follow if they experience psychiatric problems are explained.

Longitudinal studies of patients with 22q11.2DS are still rare and based on small samples, however, possible predictors of risk of psychosis are beginning to emerge (see Refs. [16,17]), and this may in the future lead to patient-tailored approaches to counselling (although more so for older children than very young patients).

Martin et al. found that almost half (48%) of genetic counsellors were uncomfortable with discussing psychiatric disorder [13]. Perceived barriers included lack of knowledge about these conditions and their treatment, as well as the stigma associated with mental disorder. Findings by Hercher and Bruenner indicated that stigma is also a concern for parents, with 42% reporting they had kept information about psychiatric problems associated with 22q11.2DS from other people for fear of stigmatisation, even though the majority of parents in this study (72%) also indicated this had rarely or never happened [12]. These findings suggest a need for training for genetic counsellors about mental health conditions and their treatment as well as the best ways to divulge this information, whilst aiming to reduce parental anxiety about societal bias with regards to these conditions. Furthermore, the follow-up sessions we propose would help parents consolidate and add to their understanding of mental health issues associated with their child's genetic syndrome.

Our study found that the internet was the most frequently reported source of information about psychiatric disorder (41–44% of parents), which is lower than the figure of 62% reported by Hercher and Bruenner [12]; however, no direct comparison is possible because we asked about parents' first source of information, while Hercher and Bruenner asked about all sources and allowed parents to endorse all options that applied.

Many CNV conditions, such as 22q11.2DS are rare; one parent wrote “Most of the information I got came from … the internet, simply due to most medical professionals having never come across this disorder before”. The internet can be an important first port of call, or additional source of information, provided the website is based on appropriately evaluated clinical and scientific information. One parent left the following comment as part of our survey: “Too little, too late. In this modern age of technology, please give parents approved websites in order to get valid information”.

However, it is also important to recognise that an internet resource cannot replace the interaction with a clinical professional. Another parent commented: “We were pointed at the internet and left to find out for ourselves”.

In addition to the development of high-quality websites to which parents can initially turn, mechanisms need to be in place to ensure that concerned parents know how to seek clinical advice and talk to well-informed clinicians.

## Figures and Tables

**Table 1:** Parental awareness (UK) and first source of information of manifestations of del22q11.2

Parents reporting awareness		Physical			Developmental		Psychiatric			
Palate defects	Heart defects	Kidney defects	Learning difficulties	Speech delay	Obsessions/compulsions	Psychosis/schizophrenia	Depression	Anxiety
51/54 (94.4%)	50/54 (92.6%)	45/54 (83.3%)	54/54 (100%)	53/54 (98.2%)	37/54 (68.5%)	46/54 (85.2%)	44/54 (81.5%)	42/54 (77.8%)
Information from clinicians	At diagnosis in clinic	20 (39.2%)	25 (50.0%)	11 (24.4%)	16 (29.6%)	20 (37.7%)	4 (10.8%)	4 (8.7%)	4 (9.1%)	4 (9.5%)
Later by geneticists/doctors	10 (19.6%)	7 (14.0%)	17 (37.8%)	11 (20.4%)	9 (17.0%)	5 (13.5%)	10 (21.7%)	8 (18.2%)	6 (14.3%)
Information from other sources	Internet	13 (25.5%)	6 (12.0%)	11 (24.4%)	18 (33.3%)	12 (22.6%)	16 (43.2%)	20 (43.5%)	19 (43.2%)	17 (40.5%)
Friend/support groups	1 (2.0%)	1 (2.0%)	0 (0%)	1 (1.9%)	0 (0%)	5 (13.5%)	6 (13.0%)	6 (13.6%)	6 (14.3%)
Books/leaflets	2 (3.9%)	1 (2.0%)	4 (8.9%)	4 (7.4%)	3 (5.7%)	3 (8.11%)	4 (8.7%)	4 (9.1%)	4 (9.5%)
Other	5 (9.8%)	10^a^ (20%)	2^a^ (4.4%)	4 (7.4%)	9 (17.0%)	4 (10.8%)	2 (4.4%)	3 (6.8%)	5 (11.9%)

a Includes when physical health condition was diagnosed before the 22q11.2 deletion.
